# A systematic review of deep learning techniques for apple leaf diseases classification and detection

**DOI:** 10.7717/peerj-cs.2655

**Published:** 2025-01-31

**Authors:** Assad Souleyman Doutoum, Bulent Tugrul

**Affiliations:** 1 Computer Science Department, University of N’djamena, N’djamena, Chad; 2 Deparment of Computer Engineering, Ankara University, Ankara, Türkiye

**Keywords:** Deep learning, Apple, Leaf diseases, Classification, Detection

## Abstract

Agriculture sustains populations and provides livelihoods, contributing to socioeconomic growth. Apples are one of the most popular fruits and contains various antioxidants that reduce the risk of chronic diseases. Additionally, they are low in calories, making them a healthy snack option for all ages. However, several factors can adversely affect apple production. These issues include diseases that drastically lower yield and quality and cause farmers to lose millions of dollars. To minimize yield loss and economic effects, it is essential to diagnose apple leaf diseases accurately and promptly. This allows targeted pesticide and insecticide use. However, farmers find it difficult to distinguish between different apple leaf diseases since their symptoms are quite similar. Computer vision applications have become an effective tool in recent years for handling these issues. They can provide accurate disease detection and classification through massive image datasets. This research analyzes and evaluates datasets, deep learning methods and frameworks built for apple leaf disease detection and classification. A systematic analysis of 45 articles published between 2016 and 2024 was conducted to evaluate the latest developments, approaches, and research needs in this area.

## Introduction

Agriculture plays a vital role in the world economy since it expands the global economy and ensures food security. The Food and Agriculture Organization of the United Nations estimates that up to 40% of global crop production is lost as a result of plant pests and diseases each year (Food and Agriculture Organization, 2024). However, apple farming plays a crucial role in the global farming industry, giving farmers worldwide a sizable source of income and supplying consumers with a wide range of apple products (Kumar, Nelson & Gomathi, 2024). One of the world’s most nutrient-dense and beneficial fruits, apples are high in dietary fiber, antioxidants, vitamins, and a range of minerals that prevent several illnesses. Production and consumption of apples are rising along with the demand for these fruits (Li, Zhu & Che, 2024b). Each year, apple production surges in response to increasing demand. A large number of apple farms, both big and small, produce an abundance of apples each year. As a result of limited equipment and lack of professional staff, small farms receive little support in keeping their fruits free from infections. This makes it difficult for them to maintain healthy crops and ultimately reduces their productivity and profitability. They therefore need efficient and cost-effective technological support to maintain healthy crops and remain profitable against large farms. In addition to reducing fruit yield, diseases also negatively affect fruit size and quality (Kumar, Kumar & Gupta, 2022). Therefore, preventing and treating apple leaf diseases is crucial to benefit both farmers and consumers.

Apple leaf diseases are frequently caused by fungi (apple scab, rust, gray leaf spot, powdery mildew), bacteria (fire blight), or viruses (apple mosaic virus). Apple scab causes black, velvety dots on the leaves, whereas rust causes orange or yellow spots, which can lead to leaf loss. Gray leaf spot can cause black lesions, whereas powdery mildew produces a white, powdery growth on leaf surfaces. Apple mosaic virus generally causes mottled or patchy discoloration on the leaves, resulting in a mosaic-like pattern. Fire blight is characterized by blackened, withered leaves that may appear charred as if burned by a fire. However, since the visual features of some diseases are the same and it is not always easy to distinguish between different phases of the same sickness, there could be a significant disparity in the diagnostic results (Devi, 2023). Apple leaf diseases can spread to fruits, leaves, branches, roots, and other tree parts. Although they often originate in the leaves, they are the easiest to spot, gather, and treat. As a result, they are a valuable resource for identifying diseases, and efficient computerized disease detection is necessary (Di & Li, 2022). The primary strategy for managing leaf diseases is agricultural pesticides. However, conventional methods of applying agrochemicals disregard the severity of the disease and employ a constant dosage. This results in inadequate dosages in some places and too high in others. Insufficient agrochemical application impedes disease prevention and mitigation. In reality, producers have to manually determine the type of spot and assess the severity of the infection (Zhu et al., 2023a). However, because appearance is subjective, errors are possible (Jiang et al., 2019). To ensure that agricultural pesticides are applied accurately, it is vital to use computers and technology related to artificial intelligence. This will help producers assess the impact of diseases (Zhu et al., 2023a).

Symptoms associated with leaves are a valuable resource for diagnosing diseases in various fruit plant species (Khan et al., 2019). Miscellaneous image processing procedures, including acquisition, filtering, segmentation, and feature extraction, are employed to identify and categorize leaf diseases and quantify various leaf measures (Gargade & Khandekar, 2019). Digital image processing techniques improve the likelihood of early plant disease detection so that necessary preventive measures can be implemented (Yadav, Akanksha & Yadav, 2020). As a result, implementing an automated computerized system for the early identification and categorizing of apple leaf evidence is imperative (Kaur & Chadha, 2023).

Numerous methods for identifying illnesses have been created in the past ten years to avoid significant losses. Methods from immunology and microbiology allow for accurately determining causal agents (Yadav, Akanksha & Yadav, 2020). These methods aim to identify a variety of diseases at an early stage so that proper care can be administered on schedule. Most of these methods divide images into several pre-established classes using computer vision and deep learning. Recent advances in machine learning have resulted in a subset of research known as deep learning (DL). DL models use a hierarchy of several layers to learn (multiple levels of) representation (Khan, Quadri & Banday, 2021). Recently, advanced deep learning techniques called convolutional neural networks (CNNs) have been applied to computer vision applications, particularly those involving image categorization. CNNs, however, can discern between crucial traits and others. Higher agricultural and crop management quality is made possible by the very high accuracy that DL’s application in plant disease diagnosis and agriculture has demonstrated (Nunoo-Mensah et al., 2022).

**Figure 1:**
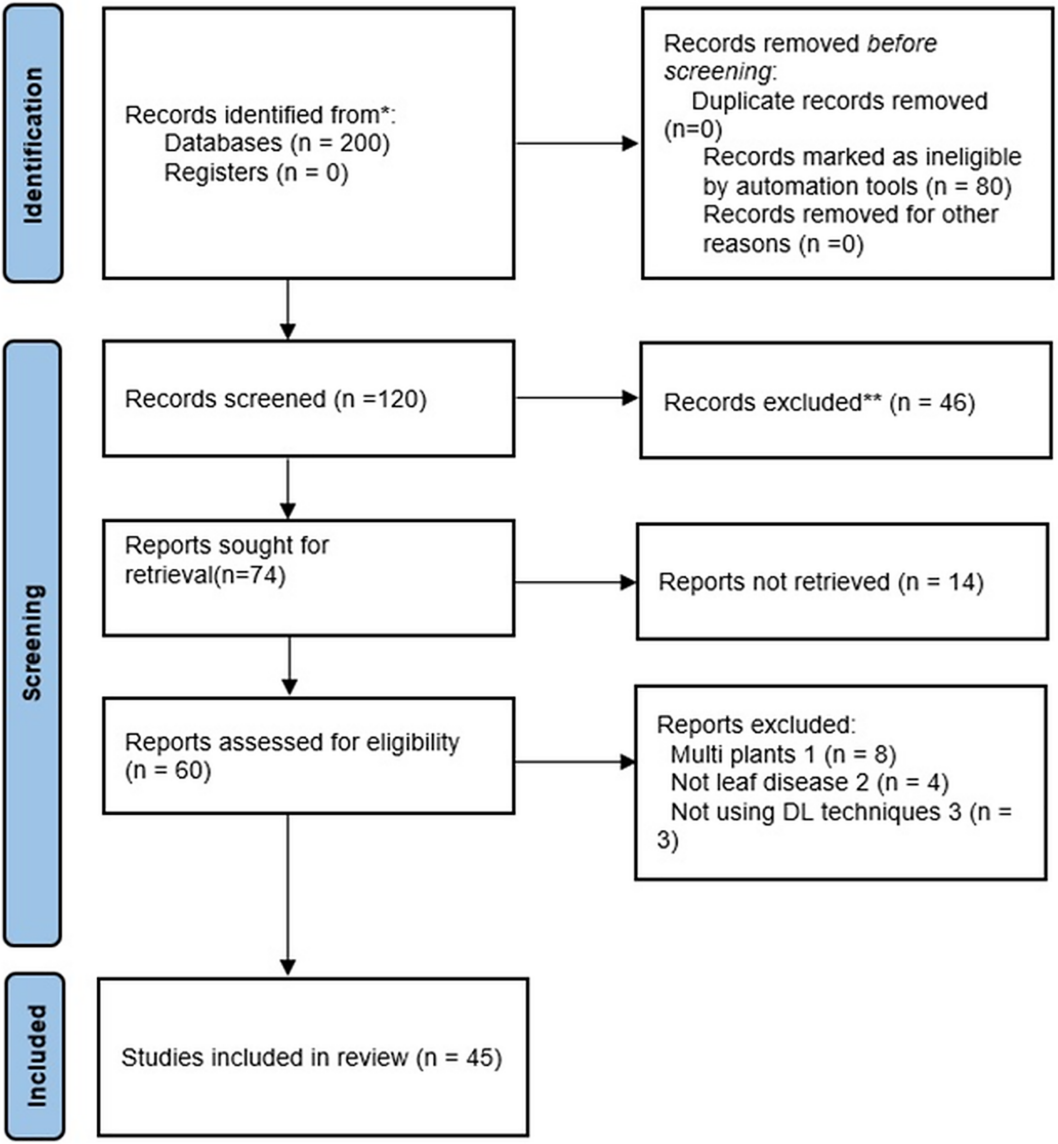
PRISMA flow chart.

Our objective is to provide an in-depth analysis of deep-learning methods for identifying diseases in apple leaves. To help readers comprehend the use of deep learning techniques for apple disease diagnosis, the review documents every relevant proposal published between 2016 and 2024. Figure 1 shows a schematic diagram of the article selection process. This article is broken down into several sections. “Deep Learning” and “Convolutional Neural Network” discuss DL and CNN architecture and its subcomponents. “Materials and Methods” presents literature review planning. We introduced apple leaf diseases in “Taxonomic Structure of Apple Leaf Diseases” In “Datasets”, we analyze the sources of miscellaneous Apple image datasets. “Deep Learning Techniques for Detecting Apple Leaf Diseases” explains DL techniques applied to apple leaf diseases. “Challenges Facing DL Techniques and Prospective Paths for Further Studies” examines DL techniques’ challenges and future work directions. “Discussion” presents a discussion and the last section provides conclusions.

### Deep learning

Machine learning (ML) and deep learning (DL) are types of artificial intelligence (AI) that learn from data and use algorithms to make decisions. They can analyze large amounts of data and identify patterns, allowing computers to make decisions based on those patterns. With DL, cutting-edge solutions are now available in a wide range of computer vision fields. Several traditional methods were proposed to recognize plant diseases before deep learning, including decision trees, random forests, and support vector machines (Doutoum & Tugrul, 2023). A DL model learns representations in a hierarchy of multiple layers. As a primary advantage, deep learning methods are independent of manually extracted features (Khan, Quadri & Banday, 2021). DL has enabled recent breakthroughs in computer vision, opening the door to dependable and effective visual systems widely employed in various fields, including robots, autonomous vehicles, and medical image analysis (Upadhyay & Gupta, 2024). Leaf diseases can be accurately identified through DL as well. An individual model can accomplish several tasks using multi-task learning (Jiang et al., 2021).

Many DL architectures are available, including AlexNet, LeNet, ResNet, MobileNet, and GoogLeNet. The design parameters of these models, such as the number of units, depth, learning process, *etc*., vary. Often, the number of layers, namely, convolution, pooling, and fully connected layers, can be adjusted to achieve the appropriate level of precision in the outcome based on the complexity of the problem. On the other hand, managing a deeper structure becomes more complex and increases the amount of processing carried out by the network. Another well-known characteristic of deep models is that they need much data to provide better outcomes (Chouhan, Kaul & Singh, 2019). Diverse datasets help ensure robust models perform well in real-world scenarios by covering various situations and reducing biases. Models with deep learning tend to require larger data sets and more complex structures, which makes them harder to manage and use.

### Convolutional neural network

**Figure 2:**
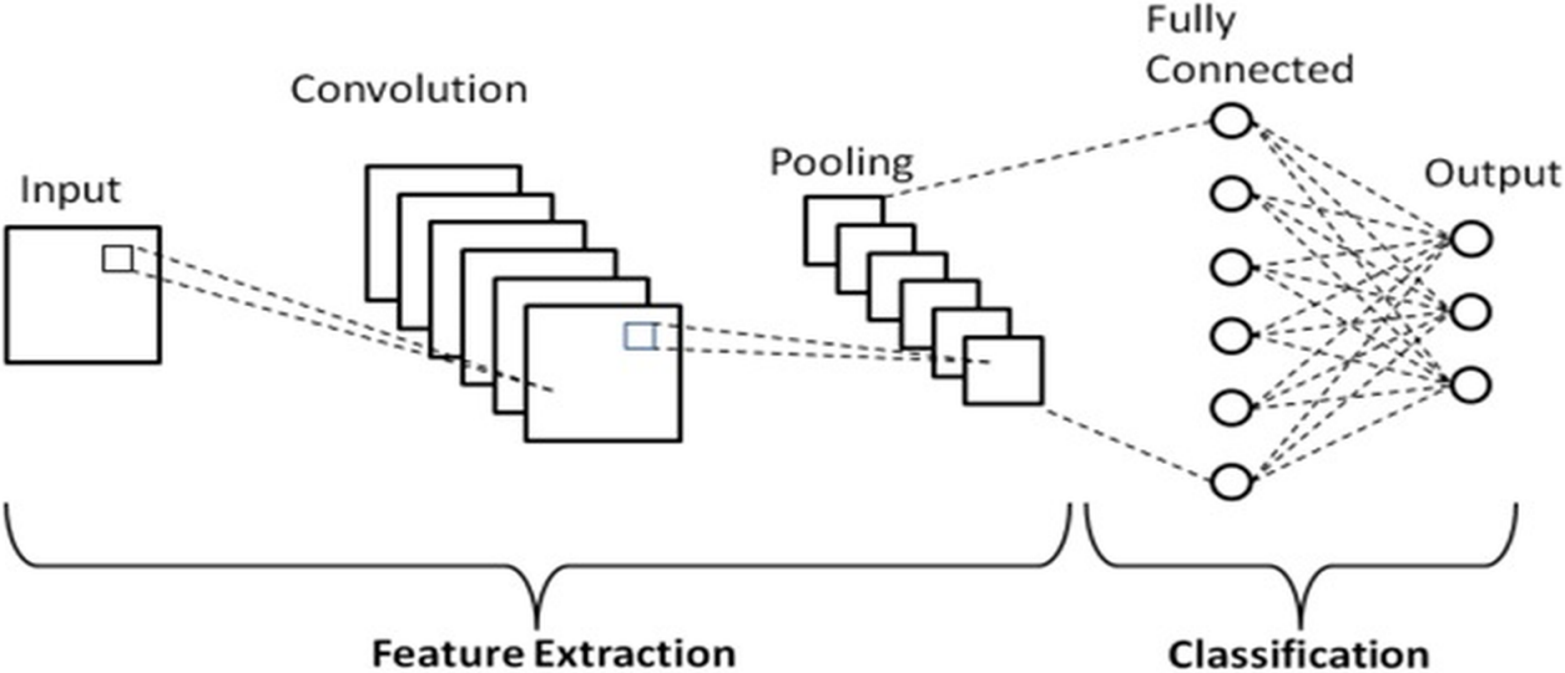
A general CNN architecture.

The most common, effective, and fundamental DL algorithm is CNN. CNN is an algorithm that learns independently; its main characteristics are expansion and adaptability. Furthermore, it has resulted in some extraordinarily significant advancements in computer vision (Haruna et al., 2023). CNN architecture is made up of multiple layers of various kinds. It usually starts with one or more convolutional layers, then moves on to one or more activation layers, grouping layers, and finally, completely linked layers. The activation function receives the output of the convolution operation, which is carried out at the convolution layer to extract features. As long as the feature map’s size is lowered, robust learning outcomes are obtained using the clustering layer (Marzougui, Elleuch & Kherallah, 2020). Therefore, convolutional, pooling, activation functions, and fully connected layers comprise its four primary layers. Figure 2 displays a general CNN architecture.

#### Convolutional layer

CNN incorporates many convolutional layers, which are critical to its performance. Convolution can be defined as a translation-invariant linear procedure where the input data is subjected to an accustomed weighting combination (Albogamy, 2021). Multiple convolutional filters, often called kernels, are used in its construction. Applying these filters to the input image, provided as N-dimensional metrics, produces a feature map (Singh, Goyal & Chandel, 2022). Therefore, choosing the correct kernel is essential to extracting the most significant information from the input data signal. This enables the model to draw more accurate conclusions concerning the data signal’s substance (Albogamy, 2021). It computes as in (1) for an input x of the ${i^{th}}$ convolutional layer. Each layer’s input (x) in a CNN model is arranged in three dimensions: depth, width, and height, or m $\times$ m $\times$ r, with width and height (m) being equal. Another name for depth is channel size. For instance, a photo’s depth (r) with RGB values is three. Furthermore, the kernels serve as the foundation for the local connections, which are convolved with input and share identical parameters ( ${W^k}$ weight and bias ${b^k}$) to generate k feature maps ${h^k}$ with a size of (m-n-1) (Alzubaidi et al., 2021).

(1) $${h^k} = f({W^k}*x + {b^x}).$$

#### Pooling layer

The pooling layer is often positioned after the convolution layer. This layer reduces the size of the output matrix generated by the convolution layer. While any size filter can be used in the pooling layer, 2 × 2 size filters are generally considered the most appropriate size (Sardogan, Tuncer & Ozen, 2018). Using 2 × 2 filters in the pooling layer notably reduces model computational complexity and memory requirements (Szeliski, 2022; Bishop & Bishop, 2023). This smaller filter size also ensures significant features are preserved while reducing dimensions, leading to more efficient processing. Additionally, 2 × 2 filters strike an appropriate balance between retaining relevant details and faster training times. Using 2 × 2 filters is like looking through a magnifying glass to focus on a small area of a larger image. It reduces the amount of data to be processed while still providing enough detail to identify what is significant. In contrast, larger filter sizes, such as 3 × 3 or 5 × 5, may capture more contextual information but come at the cost of increased computational demand and memory usage. They can also introduce more noise into the feature maps, making it more challenging to isolate the most critical aspects of the data.

**Figure 3:**
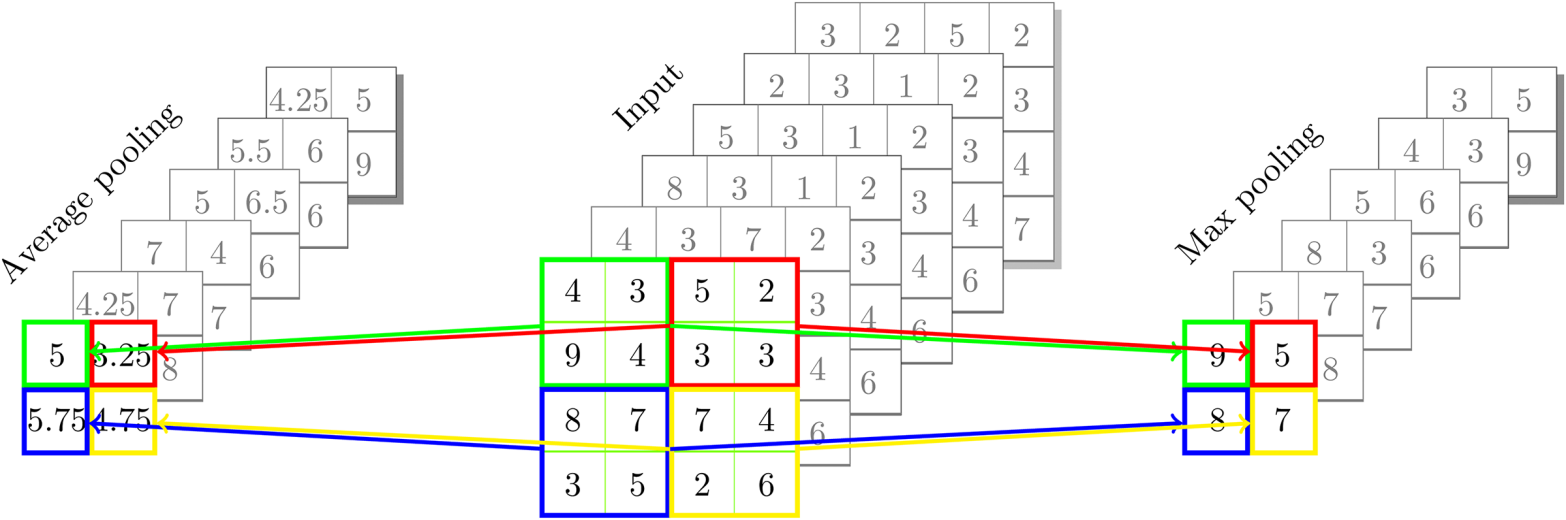
An example of pooling process.

There are two types of pooling: maximum and average, which extract the largest or average value from each subregion inside the filter’s bounds (Francis & Deisy, 2019). Maximum pooling selects the highest value from each subregion, which helps in retaining the most prominent features and is beneficial for capturing sharp and distinct patterns. In contrast, average pooling computes the average of all values in the subregion, providing a smoother representation that can be useful for reducing noise and preserving general trends in the data. Each method has its own advantages, and the choice between them depends on the specific requirements and characteristics of the task at hand. An example of a pooling process is shown in Fig. 3.

#### Activation layer

The specific diagnosis is that the activation layer activates the convolutional layer that has been extracted. It is required to include an activation layer (a non-linear function) to render the convolution process using non-linear mapping since it performs the equivalent linear transformation on the input image and the convolution kernel (Albogamy, 2021). Although there are a variety of activation functions, like hyperbolic tantric, sigmoid, and linear, rectified linear unit (ReLU) activation function is typically utilized in CNNs. The ReLU function defined by

(2) $$Relu(x) = max(0,x)$$

is a popular choice for CNNs because it has no saturation point, so the output values can be positive or negative, resulting in higher accuracy in classifying the data. Additionally, ReLU is computationally efficient, making it more suitable for deep learning networks. However, ReLU has some potential drawbacks, such as the “dying ReLU” problem, where neurons can become inactive and stop learning if they output a negative value. This can result in a loss of model capacity and hinder training progress. Additionally, ReLU can be sensitive to the choice of learning rate, which may lead to unstable training or slow convergence if not properly tuned. Overall, while ReLU is an effective and computationally efficient activation function, it requires careful tuning in order to maximize its benefits.

#### Fully connected layer

Once the convolution, pooling, and activation operations are complete, the final matrix is sent as input through the fully connected layer. In this layer, categorization and recognition are formed (Sardogan, Tuncer & Ozen, 2018). This indicates that a single vector is created by flattening the output from the previous levels. The vector’s values indicate the likelihood of each class label (Kaur & Bhatia, 2019). Therefore, the output layer is mainly set up to output the desired outcome, whereas the fully connected layer generally refits the features to minimize feature information loss (Albogamy, 2021). Minimizing feature information loss is crucial because it ensures that the essential characteristics of the input data are preserved throughout the network. This preservation allows the model to make more accurate predictions by retaining intricate patterns and relationships in the data. Consequently, reducing information loss can significantly enhance the model’s ability to generalize to unseen data, improving its overall performance. Ultimately, this results in more accurate and efficient models that can be applied to real-world scenarios.

## Materials and Methods

In this section, we will discuss how we plan to conduct the literature review and how we will revise our plan. We aim to enhance discovery reliability and trustworthiness by mitigating bias *via* an accurate review process. We also outline the prerequisites and motivation for this literature review. Moreover, effective outcome processes are significant elements of literature reviews. Effective outcome processes involve systematically evaluating and synthesizing existing research to draw comprehensive conclusions. This includes categorizing studies based on their methodologies, identifying gaps in the literature, and assessing the quality and relevance of the findings. By doing so, we ensure that our review provides a solid foundation for future research and practical applications.

### Research strategy

**Figure 4:**
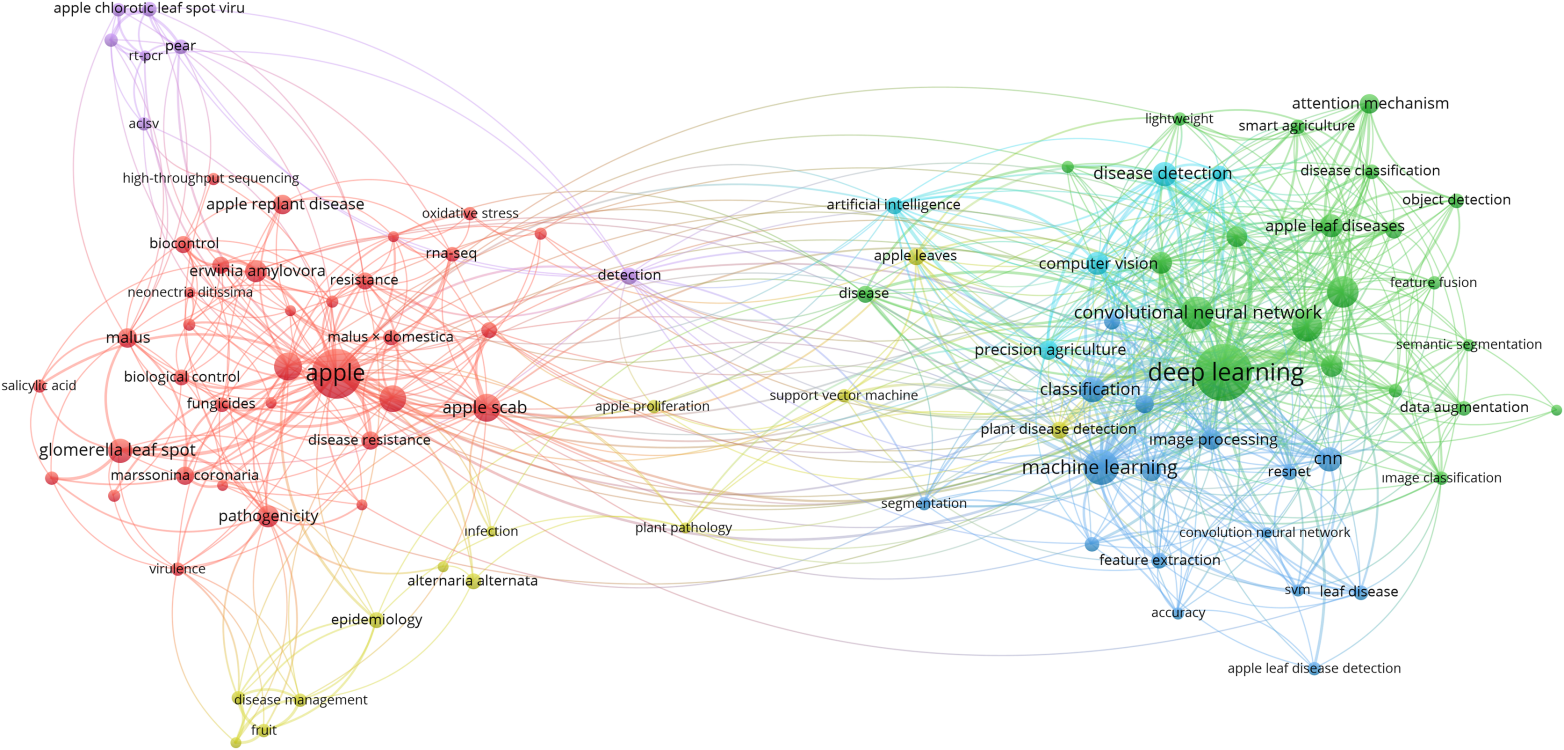
Bibliometric network visualization of keywords associated with apple leaf diseases.

This literature review examines pertinent research findings and applies them to apple leaf diseases. Articles about apple leaf diseases are chosen based on machine learning and deep learning. Figure 4 shows the different sources we used for our search databases. These databases include conference articles and journal articles. Choosing suitable databases is crucial because it ensures a comprehensive literature review and consists of the most relevant and up-to-date research findings. Access to a wide range of sources allows for a more accurate understanding of the current state of knowledge in the field. Moreover, selecting well-respected and peer-reviewed databases enhances conclusions’ credibility and reliability.

We examined several studies that used deep learning techniques to identify apple leaf diseases. In this review, apple leaf diseases, deep learning, detection, and classification are the key search terms. Every principal phrase was characterized by synonyms. We used the Boolean operators “AND” and “OR” in our search parameters. Search engines can restore correlations between apple leaf diseases and identification and categorization. By combining the key search terms with logical operators, we created a search query that returned results related to the topics we desired. This allowed us to access a comprehensive set of studies related to apple leaf diseases. Furthermore, search engines can analyze the connections between these different topics, providing a more thorough overview of the research in the area.

Figure 4 shows a bibliometric network visualization that represents clusters of keywords commonly associated with apple leaf diseases in the literature. Nodes represent keywords, and their sizes indicate their frequency. The connections (edges) between nodes reflect co-occurrences in the dataset and color coding identifies ten clusters.

### Literature review search selection criteria

**Figure 5:**
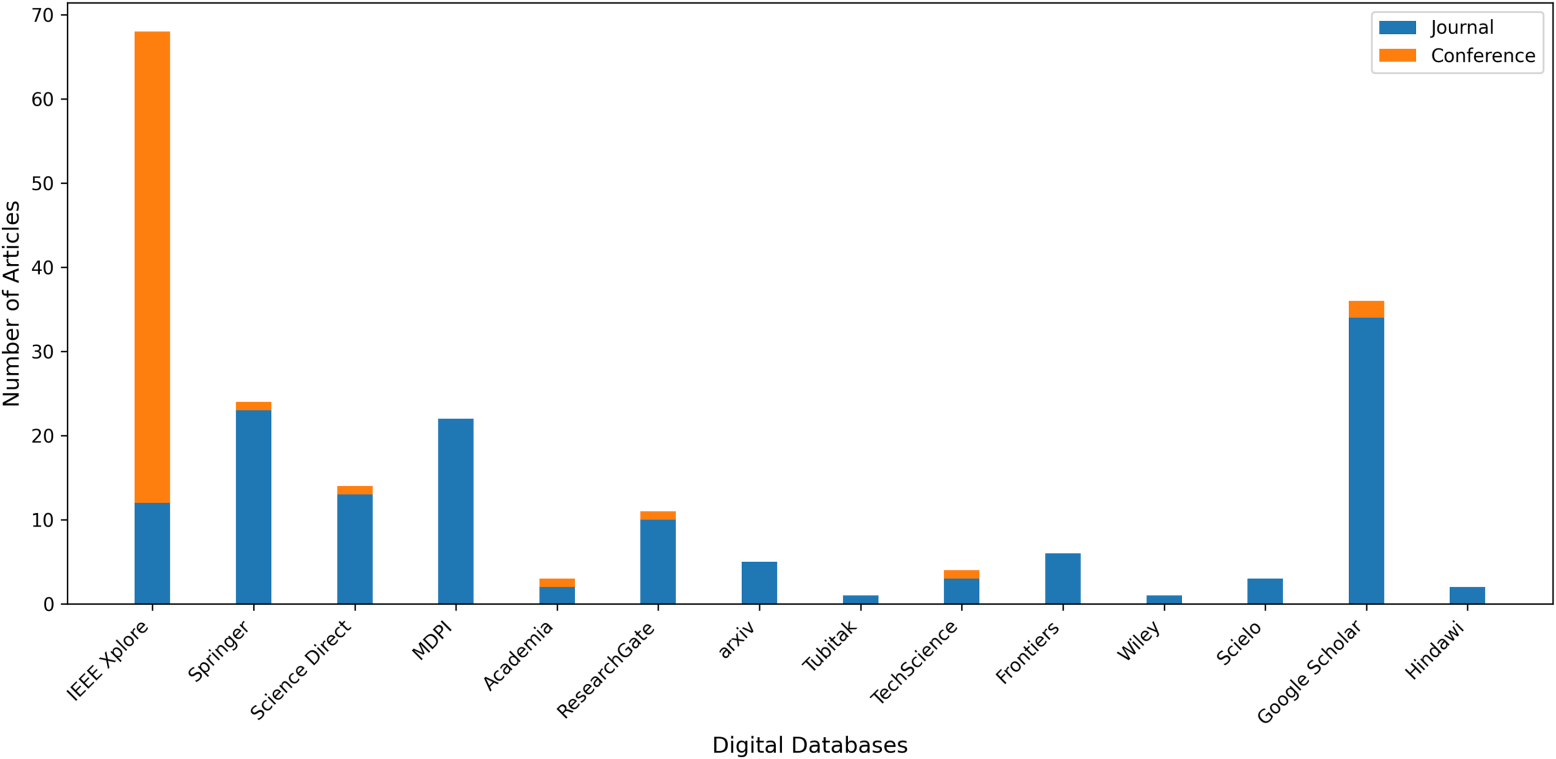
Number of articles retrieved from various digital databases.

**Figure 6:**
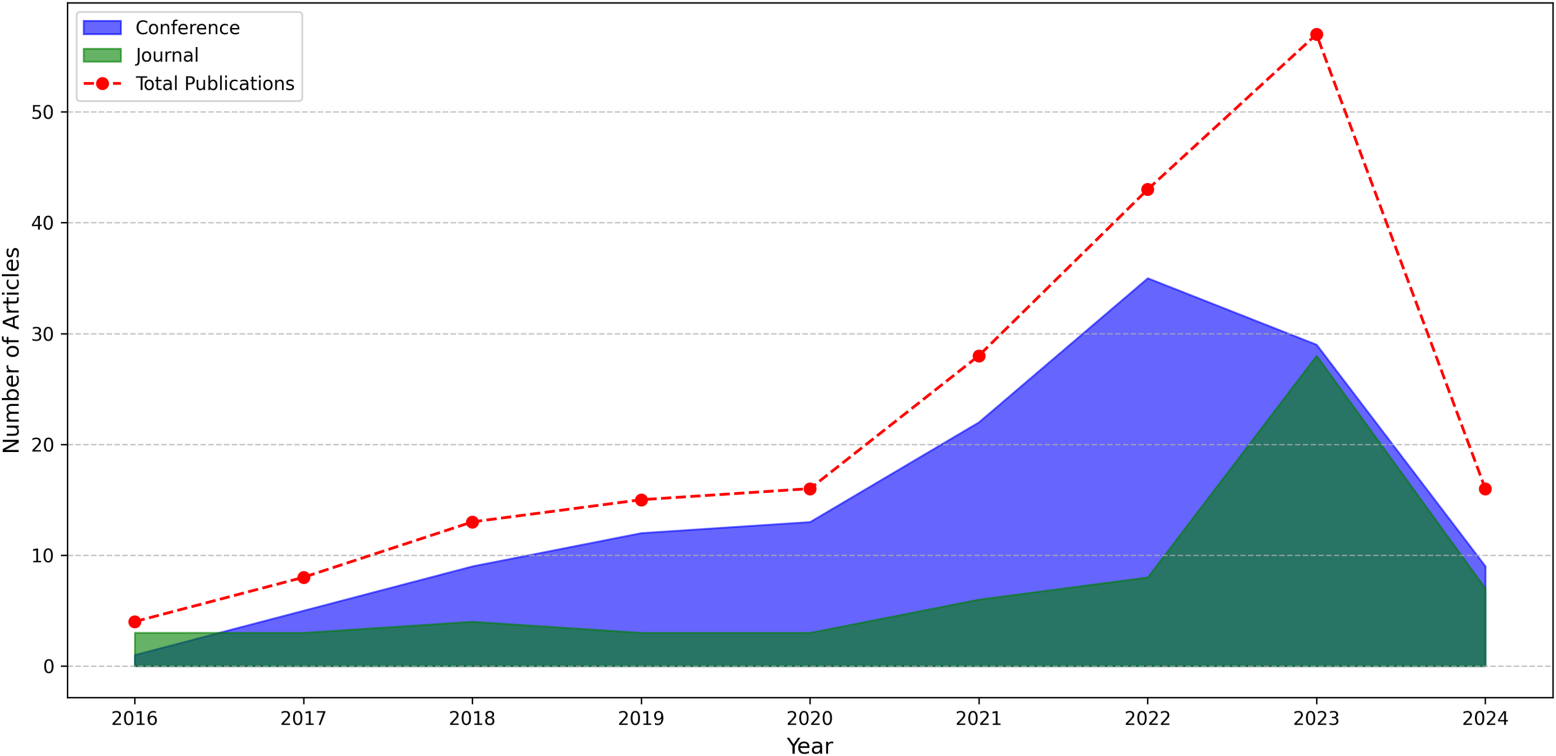
Number of conference and journal articles published between 2016 and 2024.

Our query selection standards explain how we select relevant content from search results. We aim to find all relevant research articles on our subject that address our research questions. Figure 5 displays those electronic databases and the number of publications selected from each source. The articles were organized into articles, conferences, and workshops. The IEEE Xplore database contains the most articles with an attention rate of 34.5% (69). Google Scholar is in second place with 18% (36). Springer follows them with a ratio of 12% (24). Initially, 200 studies were selected from a variety of databases. Several of them were redundant and unrelated to our research requirements and had to be eliminated. Figure 6 presents both number of conference and journal articles published between 2016 and 2024 covered by the analysis.

### PRISMA flow chart

The Preferred Reporting Items for Systematic Reviews and Meta-Analyses (PRISMA) diagram provides a clear and standardized way to report the flow of information through the different phases of a systematic review. This helps researchers and readers understand which studies were included, excluded, and why, ensuring transparency and reproducibility. Additionally, it enhances the review’s credibility by visually summarizing the selection process. Figure 1 depicts the article selection criteria. Only English-language publications published between 2016 and 2024 were selected for this study. Articles that do not qualify for this requirement are excluded. For the purpose of identifying selected studies, the following procedures are used:

#### Identification

As a starting point, articles were compiled using different techniques, including manual searches and literature databases, such as Google Scholar, IEEE, Springer Link, Science Direct, Frontiers, Research Gate, Hindawi, and MDPI, *etc*. Drawing from a wide range of sources ensures a comprehensive understanding of the topic by incorporating various perspectives and methodologies. This diversity enriches the research by highlighting different findings and theories, ultimately leading to more robust and well-rounded conclusions. Additionally, utilizing multiple databases and platforms helps uncover less visible or niche studies that might otherwise be overlooked. To begin with, two hundred publications pertinent to our study were selected in total.

#### Screening

Screening procedures ensure that only the most pertinent and high-quality literature is included in a study. They help filter out irrelevant or redundant articles, allowing researchers to focus on the most impactful and reliable data. The total number of records is shown after duplicates are eliminated. The titles and summaries of publications are checked for relevance to the study’s topic. Duplicate articles and publications were removed. After the screening procedure, forty six journal articles were excluded.

#### Eligibility

The PRISMA diagram displays the total number of research articles that moved to the next stage after initial screening. Full-text publications of potentially relevant research are evaluated in light of qualifying requirements. Therefore, we scanned and examined sixty publications to verify the results. Fifteen studies were removed due to their lack of significance or irrelevance concerning research topics and debates.

#### Inclusion/exclusion

It provides an overview of all research publications included in our thorough analysis. Therefore, these articles meet the established eligibility conditions and are considered suitable for additional examination. The flow chart displays the number of studies excluded at each stage and the justifications for those selections. Exclusion is often due to factors such as inadequate study design, lack of information, or inability to meet certain inclusion conditions. The forty-five articles justified this comprehensive evaluation.

### Taxonomic structure of apple leaf diseases

One of the world’s healthiest and most nutrient-dense fruits, apples are loaded with vitamins, minerals, fiber, and antioxidants. Apple diseases harm the quantity and quality of its fruit (Kumar, Nelson & Gomathi, 2024). Three different categories of apple diseases exist: bacterial, viral, and fungal infections (Gorretta et al., 2019). They can cause various symptoms, including wilting, discoloration, stunted growth, and premature plant death. The soil or wounds can be potential entry points for them to penetrate the body (Charan & Mohammed Abrar, 2022). These diseases can lead to significant losses in apple production by reducing yield and affecting fruit quality. Therefore, it is important to be aware of these diseases and take preventive measures to reduce the impact of their infections. Table 1 illustrate how several pathogens can cause these conditions.

**Table 1:** List of apple leaf disease types, symptoms, causes and image.

Disease type	Symptoms	Effects
Apple scab	The discharge of ascospores starts the main infection. A flat mycelium forms between the cuticle and the epidermal cell walls due to these spores penetrating the leaf cuticle (Rexhepi, Paçe & Vrapi, 2017).	One of the most harmful infections that impact apple productivity is the fungus *Venturia inaequalis*, which is the cause of apple scab (Bracino et al., 2020).
Black rot	On affected branches, cankers appear as a deep, reddish-brown patch. Canker commonly has rough, fractured bark (Kumar, Kumar & Gupta, 2022).	The tree becomes weaker due to droppings, and girdling cankers lead to harm and stem decline (Kumar, Kumar & Gupta, 2022).
Apple rust	The kidneys or round-shaped galls are light brown, reddish brown, or chocolate brown in color (Biggs et al., 2009).	Pucciniaceae glue rust infections harm apple leaves and fruit (Liu et al., 2017). A fungus that attacks both apples and cedars causes a disease known as apple rust, which is a fitting moniker (Stone, 1908).
Powdery mildew	Small patches of white or grey powdery sap show up on the leaf undersides. The leaf blades have a curled border and grow longer and thinner than regular leaves. Twigs are dusted with powder (Kumar, Kumar & Gupta, 2022).	The fruit has damaged surfaces (Kumar, Kumar & Gupta, 2022).
Alternaria	Leaves are brown in hue, spherical in form, and sometimes bordering on purple. They become ash brown to tan with time, and a few of the spots have additional enlargement that causes them to take on an uneven shape (Kumar, Kumar & Gupta, 2022).	The fungus that causes “frog eye leaf spot,” also referred to as “Black Rot,” or “Physalospora obtuse,” can kill apple plants at various stages of development (Bonkra et al., 2024).
Marssonina	Brown patches initially form on the leaf’s surface, and over time, they become dark leaves: brown in hue, spherical in form, and sometimes bordering on purple. They become ash brown to tan with time, and a few of the spots have additional enlargement that causes them to take on an uneven shape of brown (Kumar, Kumar & Gupta, 2022).	Apple Marsonina Blotch (AMB) is an undesired fungus that causes fruit and leaves to shrink, defoliate early, and create less starch (Bonkra et al., 2023).
Fire blight	This disease travels downhill from the peak to the bottom. The blooms abruptly change to brown and wither, and the foliage turns to brown, while the tips of the shoots bend (Kumar, Kumar & Gupta, 2022).	The infections can kill whole trees or only a few young branches (Kumar, Kumar & Gupta, 2022).
Mosaic virus	In apple trees, the mosaic signs ranged from moderate to serious chlorosis, bright cream-colored irregular patches and rings, and pale-yellow mosaic (Xing et al., 2018).	It reduces photosynthetic efficiency due to chlorosis and necrosis. There is a decline in tree vigor and fruit yield (Xing et al., 2018).

### Datasets

Plant improvement could proceed faster with automated detection and classification of plant diseases, making field surveillance more efficient. However, traditional machine-learning algorithms such as decision trees and K-nearest neighbors cannot distinguish between disease types or other sources of dead plant tissue in a typical plantation. This is due to different variations in illumination and orientation in images obtained at various times and weather conditions (Doutoum, Eryigit & Tugrul, 2024). Deep learning systems need high-quality human-generated training data to identify a particular disease from field images precisely (Nunoo-Mensah et al., 2022). However, dataset construction is a crucial thing that needs to be addressed to improve accuracy and reduce overfitting and underfitting problems (Bi et al., 2022). More effort should be put into collecting, standardizing, and annotating data to enhance deep learning systems’ performance. Therefore, we examine various Apple datasets and their sources widely referenced in academic studies in this section.

**Figure 7:**
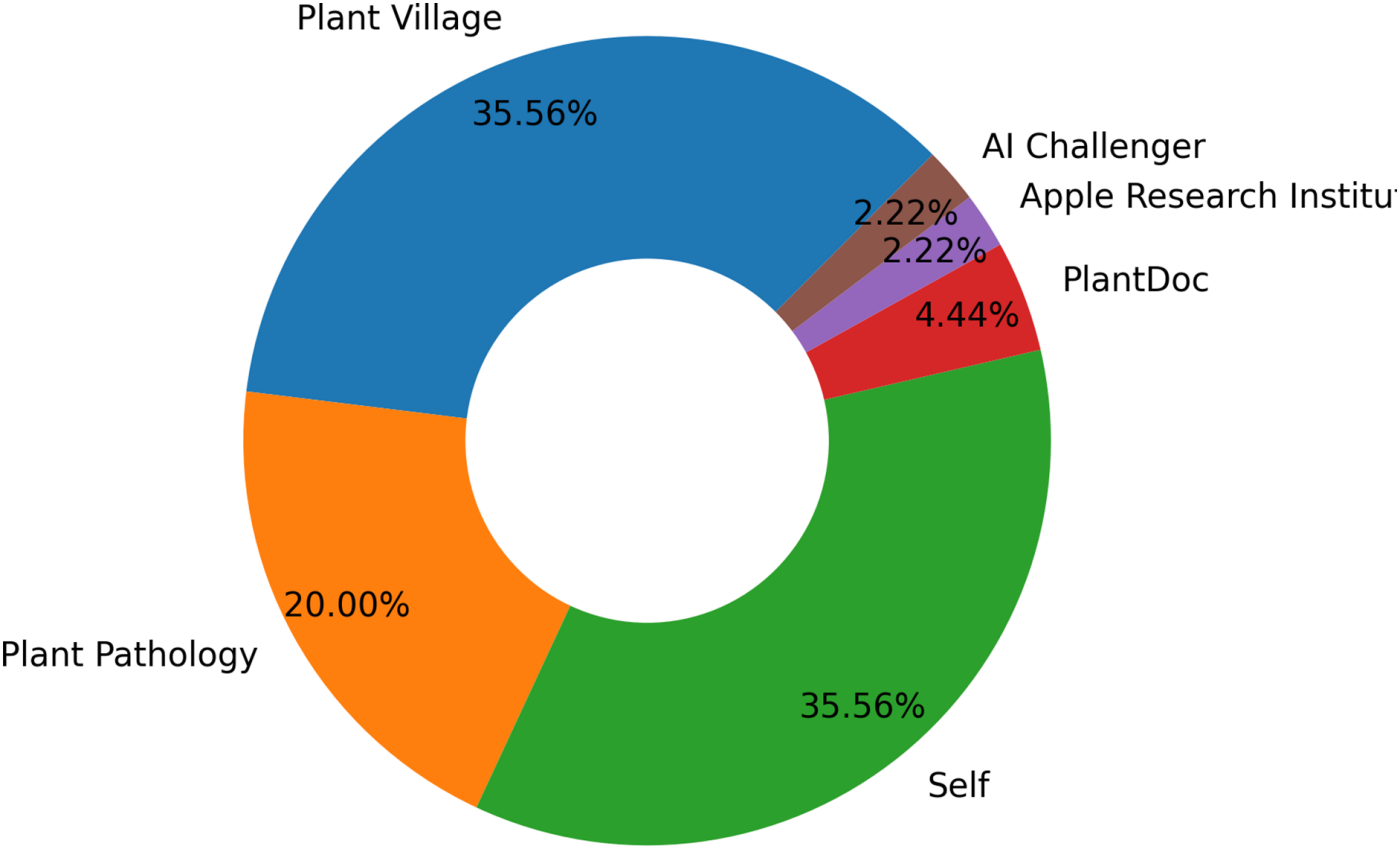
Distribution of datasets for apple leaf disease detection and classification.

The most significant public archive of leaf images is Plant Village (Hughes & Salathé, 2015). It is a not-for-profit project managed by EPFL in Switzerland and Penn State University in the USA. There are 54,309 images in the collection, representing 14 different plant species and 38 classes of healthy and unhealthy leaves (Nunoo-Mensah et al., 2022). Plant pathology ranks second as a public repository of free leaf datasets (Thapa et al., 2020). The self-dataset source leads with 16 apple data sets obtained from fields. Figure 7 displays the various image datasets available to classify and detect apple leaf diseases.

## Deep learning techniques for detecting apple leaf diseases

Apple leaf diseases can be identified using various methods and strategies. This section outlines the datasets and DL techniques used and the contributions and constraints of current research DL methods. Based on this perspective, the recent studies presented between 2016 and 2024 will be evaluated. The search keywords for articles were: “apple leaf disease detection by deep learning” and “apple leaf disease classification based on deep learning”. Only one research article was published on apple leaf disease detection in 2016. In 2017 and 2018, there was an increase in the number of deep learning research. A total of four articles were published in both years. The search results indicate that eight and 12 articles were published in 2019 and 2020, respectively. This shows that articles published on apple leaf disease detection are becoming more prevalent. Deep learning methods for detecting apple leaf disease in 2021 and 2022 were 23 and 38, respectively. The number of articles published in 2023 and 2024 was 46 and 10, respectively. These observations confirm that deep learning methods are becoming increasingly popular for diagnosing apple leaf disease. They also demonstrate the effectiveness of the technique and the need for further research in this area. Future research should focus on improving the accuracy and efficiency of deep learning models for apple leaf disease detection by exploring novel architectures and techniques.

Nachtigall, Araujo & Nachtigall (2016) investigate the automatic detection and classification of illnesses, nutritional deficiencies, and herbicide-induced damage on apple trees using CNN based on AlexNet on leaf images. The dataset was collected from three species containing 2,539 leaves divided into five classes. They used the Caffe framework in the NVIDIA environment to train the dataset for 300 epochs. The AlexNet model achieved an accuracy of 97.30% (Nachtigall, Araujo & Nachtigall, 2016). Liu et al. (2017) applied AlexNet Prior as a model. The Cascade Inception architecture has two max-pooling layers and two inception components. The dataset of 1,053 images has four classes. A CNN-based model was used to control apple diseases. The total training images were 13,689 after data augmentation techniques. In the NVIDIA Tesla environment, the DL model was trained on the Caffe framework to detect apple leaf diseases with an accuracy rate of 97.62% (Liu et al., 2017).

Singh & Gupta (2018) use a four-classifier classification approach to predict diseases caused by *Marsonina coronaria* and apple scab utilizing SVM, KNN, regression tree, and naive Bayes classifiers. 900 photos were shot, of which 350 featured *Marsonina coronaria*, 350 featured apple scab illnesses, and 200 images of apple leaves free of infections. These images were gathered from various farms with a digital camera. The experiments were done in the MATLAB framework, applying ML and DL architectures. The authors used ROC and AUC techniques to measure model performance. The authors concluded that the KNN model achieved the highest accuracy of 99.40% compared with other ML classifiers (Singh & Gupta, 2018). Baranwal, Khandelwal & Arora (2019) applied a CNN-based model to automatically detect apple leaf diseases in their early stages and prevent their spread. A dataset of 2,526 images for four classes was collected from the Plant Village dataset. Data augmentation techniques in the Keras library were applied to increase the number of images in the dataset. The dataset was trained using the GoogLeNet model on the TensorFlow framework. The DL model achieved an accuracy rate of 98.42% (Baranwal, Khandelwal & Arora, 2019).

Yan et al. (2020) present an enhanced VGG16 model for diagnosing apple leaves. It is necessary to identify three major apple leaf diseases and healthy leaves. Those four classes were selected from 27 other classes from the 2008 “Al Challenger” dataset. The dataset contains 2,446 images, 1,340 healthy images, 411 scabs, 487 frog eye spots, and 208 cedar rust. The authors used global average pooling and other regularizations to reduce the number of parameters and improve the overall performance of the VGG16 model. The dataset was trained after data augmentation, such as rotation and flipping horizontally and vertically. The experiment showed that the proposed model reached an accuracy rate of 99.01% on the test set (Yan et al., 2020). Li & Rai (2020) proposed three classifiers, ResNet-18, ResNet-34, and VGG-16, to detect and identify apple leaf diseases. The dataset was downloaded from the Plant Village website (https://plantvillage.psu.edu/). It contains four categories, three diseased and one healthy leaf. Using the TensorFlow framework, the NVIDIA environment was used to train the model on 60% of the dataset. ResNet-18 model showed the highest performance compared with the other models, with an accuracy of 97.00% after data enhancement (Li & Rai, 2020).

Chao et al. (2021) pre-trained a variety of CNN models on a subset of the Plant Village dataset (https://www.kaggle.com/competitions/plant-pathology-2021-fgvc8) with Keras using the TensorFlow framework. The authors proposed SE_Xception based on Xception architecture to identify apple leaf diseases. Then the proposed model was trained on the Apple Tree Leaf Disease Dataset (ATLDD) collected from apple experimental and demonstration stations at Northwest A&F University in China. ATLDD consists of 2,975 images of five types of diseased and healthy leaves. The proposed SE_Xception model performs better than the Xception model regarding categorization accuracy. The model achieved an accuracy of 99.40% on the test set (Chao et al., 2021). An apple leaf dataset of four classes (black rot, rust, scab, and healthy leaf) from the Plant Village website was collected. The experiment was done on Keras in the NVIDIA TensorFlow framework. Transfer learning is used to pre-train the model. The overall accuracy of the proposed model was 99.12% on the test set and F1-score 99.00%, respectively (Mahato, Pundir & Saxena, 2022). Fu et al. (2022) trained a dataset on the PyTorch framework of Torchvision. The dataset contains five classes and is available publicly in the Plant Village dataset. A multi-scale convolutional neural network is added to the suggested model to handle many Apple datasets and improve overall performance. In addition, the fully connected layer was simplified to reduce the parameter size. The proposed model achieved an accuracy rate of 97.36% (Fu et al., 2022).

A faster R-CNN method was proposed to detect apple leaf diseases. The dataset was downloaded from the Plant Pathology website. It contains five different types of diseases, a total of 4,182 images. A feature pyramid network was added to recognize objects at various scales. With an efficiency of 63.1% average precision, the technique outperforms competing object detection approaches in the annotated apple leaf disease dataset (Gong & Zhang, 2023). ResNet50V2 model is used before DCNN to recognize apple leaf diseases in their early stages. The model was trained on a dataset of 9,714 images containing four categories. This dataset was downloaded from Kaggle and divided into training, validation, and testing sets. The experiment was implemented on the Google CoLab platform, and the ResNet50V2 model achieved an accuracy rate of 98.45% (Thakur, Kapoor & Saini, 2023).

CBAM was implemented to improve ResNet-101 performance. 14,582 apple leaf images were downloaded from the Plant Village dataset. Nine classes of diseases are present in the dataset, along with healthy apple leaves. The TensorFlow framework was used to train the ResNet-101+CBAM model. The ResNet-101+CBAM with data enhancement achieved the best performance compared with the ResNet-101 with an accuracy of 96.69% (Lin, 2024). DL architectures are widely utilized in apple leaf disease detection and classification. Authors use various techniques and procedures to improve DL models’ performance. These techniques include reducing hyperparameters, data augmentation, and adding CBM and CBAM attention. Many current object detection models provide an additional perspective to consider while choosing or creating a model for illness detection and classification.

**Figure 8:**
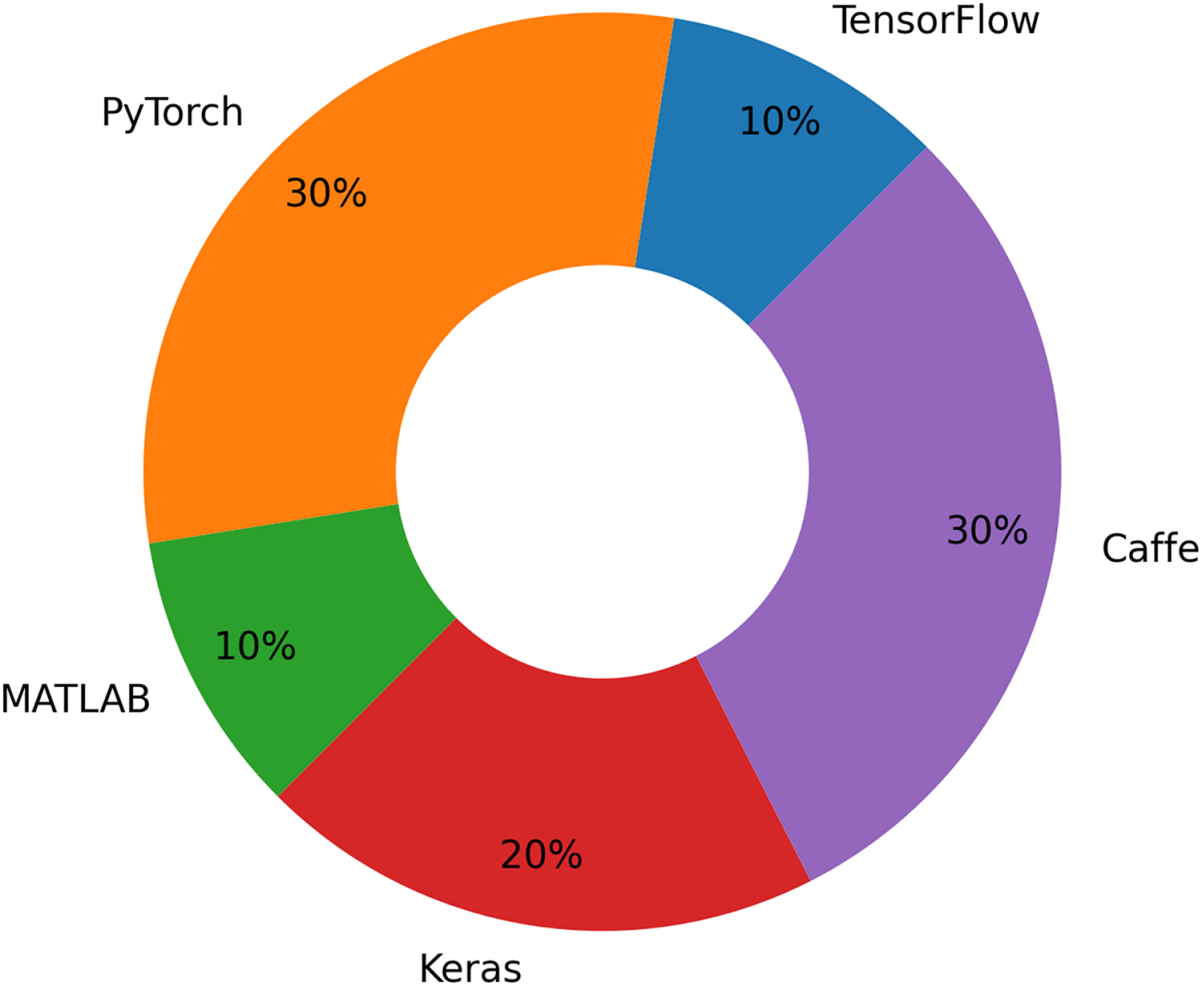
Distribution of DL frameworks for apple leaf disease detection and classification.

One option to improve recognition algorithms is AdaBoost. Identification and categorization of apple leaf diseases are the primary goals of DL methods. The study suggests that further investigation into identifying, categorizing, and measuring apple leaf symptoms can enhance intelligent farming practices. Although quantification has not received as much attention from field scientists, it can yield more informative data for quick decision-making in agribusiness. There are several DL frameworks that can be used to detect apple leaf diseases, as shown in the Table 2. Figure 8 shows the distribution of DL frameworks for apple leaf disease detection and classification. Our results conclude that the PyTorch framework is the most popular, followed by Caffe. Matlab and TensorFlow were third and fourth, respectively.

**Table 2:** DL frameworks used to detect apple leaf diseases.

Reference	# of images	DL algorithm	Framework	Performance
Nachtigall, Araujo & Nachtigall (2016)	2,539	AlexNet	Caffe	97.30%
Liu et al. (2017)	13,689	CNN	Caffe	97.62%
Singh & Gupta (2018)	900	KNN	Matlab	99.40%
Jiang et al. (2019)	26,377	INAR-SSD	Caffe	78.80%
Khan, Quadri & Banday (2021)	8,400	CNN	Keras/TensorFlow	97.00%
Wenxia et al. (2021)	81,700	CA-ENet	PyTorch	98.80%
Liu et al. (2022)	6,268	YOLOX-ASSANano	PyTorch	91.08%
Chen, Pan & Wu (2023)	1,977	CycleGAN	TensorFlow	97.78%
Li, Zhu & Che (2024b)	2,029	ResNet50	PyTorch	93.19%

## Challenges facing dl techniques and prospective paths for further studies

This section provides an overview of some challenges associated with identifying plant diseases in general and apple leaf diseases in particular. It suggests specific paths of inquiry for further study into identifying and detecting plant diseases in smart agriculture, particularly those associated with apple leaf. The articles we reviewed between 2016 and 2024 revealed some challenges with computer vision and DL techniques. These challenges include complex backgrounds and several symptom areas that are difficult to identify on leaves. Extraction of a sufficient number of features for classification, as unnecessary features affect system operation duration and do not yield enough accuracy (Saleem et al., 2022). Also, too many parameters, a slow recognition process, and poor detection efficiency in dense areas affect the model’s performance (Zhu et al., 2023b). Farmers cannot accurately distinguish between disease and dead tissues. The reason for this is that apple leaf diseases usually exhibit similar symptoms, such as browning, spots, and wilting, so it is difficult to identify the precise disease. The symptoms of the disease can often be confused by environmental factors such as drought or nutritional deficiencies. In addition, for rare or novel diseases, proper identification frequently requires laboratory testing or informed and competent individuals, which may not be available to all farmers. Therefore, agricultural practitioners need reliable AI-based solutions that accurately diagnose plant diseases with minimal human intervention (Nunoo-Mensah et al., 2022).

One approach to improving computer vision accuracy for identifying plant diseases could be enhanced image preprocessing techniques to isolate relevant features from complex backgrounds better (Chen, Pan & Wu, 2023). Additionally, advanced data augmentation methods can help increase training data diversity, enhancing model generalization (Chuanlei et al., 2017). Incorporating transfer learning from models pre-trained on similar tasks might also boost the system’s ability to recognize subtle disease symptoms more precisely. Finally, using an ensemble of models can improve system performance. Combining several models with different architectures or hyperparameters enables the detection of subtle patterns in the data that an individual model might not detect. Ultimately, these techniques could lead to more accurate predictions and help identify diseases more precisely. Table 3 summarizes the approaches and highest accuracy values achieved by studies that detected apple leaf disease from 2016 to 2024.

**Table 3:** A summary of deep learning techniques for apple leaf disease from 2016 to 2024.

References	Dataset	Method	Contribution	Best acc.
Nachtigall, Araujo & Nachtigall (2016)	Self	AlexNet	The authors created an original data set of labeled images that included illustrations of five of the most prevalent and significant illnesses affecting apple leaves.	97.30%
Chuanlei et al. (2017)	Self	SVM	The authors offered some technological know-how and a theoretical foundation for creating an automated system for diagnosing and tracking apple diseases.	93.00%
Liu et al. (2017)	Self	CNN based model	To precisely diagnose apple leaf diseases, the researchers developed a novel deep convolutional neural network model that can both automatically recognize leaf diseases’ distinctive characteristics and provide a highly accurate end-to-end learning pipeline.	97.62%
Sivakamasundari & Seenivasagam (2018)	Self	SVM	The authors presented a method for identifying diseases, such as Cedar Rust, Apple Scab, and Alternaria, in apple leaves based on their color and texture features.	92.67%
Khan et al. (2019)	Plant Village	M-SVM	The authors presented a brand-new automated technique for segmenting symptoms and choosing the optimal attributes to identify and diagnose apple leaf diseases.	97.20%
Yu & Son (2019)	Apple Research Institute	ROI-aware DCNN	The authors suggest a method for predicting ROI feature maps. Furthermore, this study takes into account some of the effects of the real environment. This is because leaf photos have complex background details.	84.30%
Aravind, PraylaShyry & Felix, 2019	Plant Village	Support vector classifier	Using gradient boosting and support vectors, the authors created a novel method for dividing apple leaf health into categories such as rot and healthy.	91.00%
Baranwal, Khandelwal & Arora (2019)	Plant Village	GoogLeNet	The authors developed a novel way to identify the type of pathogen that affects the crop. To automate this task, a convolutional neural network (CNN)-based model is trained to discriminate between healthy and infected leaf images.	98.42%
Kaur & Bhatia (2019)	Self	Deep Belief Network (DBN)	The authors presented a deep belief network-based approach trained through machine learning techniques to enable a higher and more accurate disease detection ratio.	94.70%
Agarwal et al. (2019)	Plant Village	CNN	Farmers and real-time robot designers who use the authors’ expertise to spray targeted fertilizers in fields would benefit from it. While other pre-trained models require over a minute to test, the suggested model takes less than seven seconds to do so.	99.00%
Fang et al. (2019)	Self	VGG-16	The center loss function and batch normalization are combined in the model presented by the authors. Experiments show that a modest learning rate is appropriate for training the network when selecting the initial learning rate.	97.58%
Jiang et al. (2019)	Self	INAR-SSD	The method can handle every diseased apple image from a natural apple field.	78.80%
Khan et al. (2019)	Self	CNN	By examining the diseased leaves, the scientists’ deep learning program can reliably and accurately identify and predict different kinds of apple disease.	97.18%
Yan et al. (2020)	AI Challenger	VGG-16	A global average pooling layer, a batch normalizing layer, a fully connected layer, and three completely connected layers are added to the network topology of the authors’ proposed VGG16 network.	99.01%
Li & Rai (2020)	Plant VILLAGE	ResNet-18, ResNet-34, VGG16	The authors suggested using the gray symbiosis matrix as the feature training matrix to correctly extract apple leaf features.	97.00%, 96.00%, 88.00%
Bracino et al. (2020)	Self	Gaussian Process Regression (GPR)	The authors devised a hybrid model that uses the leaf to identify the type of disease and determine whether the apple is healthy or unhealthy.	82.50%
Hameed Al-bayati & Üstündağ (2020)	Plant Village	Plant diseases detection system (PDDS)	Grasshopper Optimization Algorithm (GOA) is employed as an optimization method, Faster Robust Feature (SURF) as a feature extraction technique, and deep neural network (DNN) as a deep learning strategy.	98.28%
Yadav, Akanksha & Yadav (2020)	Plant Pathology	AFD-Net	A novel AFD-Net model using the Hybrid EfficientNet model is proposed to automate the detection and multi-classification of foliar diseases in apples.	98.70%
Slathia, Chhajer & Gouthaman (2021)	Plant Village	CNN	The suggested method segments and improves the contrast of the diseased spot.	98.00%
Reddy & Rekha (2021)	Plant Village	CNN	The authors suggested CNN be outfitted with AlexNet and GoogleNet cascades from inception as part of the Deep Leaf Disease Prediction Framework (DLDPF).	97.62%
Khan, Quadri & Banday (2021)	Self	CNN	It suggests a framework for quick and accurate disease diagnosis and ways to use predictions to improve the apple economy.	97.18%
Wang et al. (2021)	Plant Village	Coordination Attention EfficientNet (CA-ENet)	It determines the optimal parameters for network depth, width, and image resolution.	98.92%
Zhang, Yang & Cao (2021)	Plant Pathology	ResNet34	The prediction system was trained using the CNN-based model RESNET34 and implemented in the FastAI framework.	93.76%
Srinidhi, Sahay & Deeba (2021)	Plant Pathology	EfficientNetB7 DenseNet	A model with basic reduced capsules on images was suggested with only a few CNN layers.	99.80%, 99.75%
Singh, Goyal & Chandel (2022)	Self	SVM, KNN, DRT, NB	The authors proposed a KNN algorithm to predict two apple leaf diseases (apple scab and apple Marsonina coronaria).	97.20%, 99.40%, 93.10%, 62.50%
Di & Li (2022)	Plant Village	Tiny-YOLO	The Tiny-YOLO model developed by the authors demonstrated the effectiveness of their experiments and provided insight into how apple leaf diseases are detected.	90.88%
Mahato, Pundir & Saxena (2022)	Plant Village	DCNN	The suggested approach works well for object recognition and categorization in real-time. In addition, the proposed model’s performance parameters outperform those of other models.	99.31%
Liu et al. (2022)	Plant Village PlantDoc	YOLOX-ASSANano	The suggested approach enhances the network’s capacity for feature learning by utilizing the planned asymmetric ShuffleBlock. It also lowers the interference of external factors during disease feature extraction by using the suggested CSP-SA module.	91.08%
Zhu et al. (2023a)	Self	LD-DeepLabv3+	The revised two-stage model LD-DeepLabv3+ combines the Leaf-DeepLabv3+ and Disease-DeepLabv3+ models to extract apple leaves and detect diseases under a mixed background.	98.70%
Liu et al. (2023)	Self	ECA-DCMobileNet	Using a self-constructed dataset on apple leaf disease enhanced with GAN networks, the authors proposed ECA-DCMobileNet based on Mobilenet-V2. They integrated the ECA channel attention module with fewer parameters and optimal accuracy.	96.23%
Gong & Zhang (2023)	Plant Palthology	Faster R-CNN	The authors used RoIAlign rather than RoIPool, which increased detection accuracy to determine the feature map position.	63.10%
Gan et al. (2023)	Self	CNN	After data obtained from digital image processing techniques, the authors used neural networks to classify diseases.	97.60%
Tanwar, Sharma & Anand, 2023	Plant Pathology	CNN	The framework increases fruit identification accuracy in diagnosing diseases, boosting Apple’s economy by preventing health problems.	96.00%
Li et al. (2023)	Plant Pathology PlantDoc	BTC-YOLOv5s	The study examines different shapes of diseased spots to address the issue of missing and false detections.	84.30%
Nazir, Ullah & Akter, 2023	Plant Village	ResNet	This method can be used to classify a wide range of both common and uncommon apple leaf diseases.	96.21%
Upadhyay & Gupta (2024)	Self	ResNeXt	The authors proposed new model to detect apple fungal disease, transfer learning has been applied to improve accuracy results.	98.94%
Si et al. (2024)	Plant Pathology	DBCoST	The model uses CNN and Transformer to capture global information with long-range dependencies and local properties.	97.32%
Li et al. (2024a)	Plant Pathology	TPH-YOLOV5	They conducted comparative studies between three network models, YOLOV5, YOLOV5-MobileNetV3, and TPHYOLOV5.	91.40%
Kaur et al. (2024)	Self	CNN + SVM	Because of its unmatched speed and precision of detection, the model has the potential to significantly change apple disease detection.	97.08%
Math et al. (2024)	Plant Village	QBPSO	The SqueezeNet feature extraction layer performs well by independently initializing with a large activation map.	98.76%
Rawat & Singh (2024)	Plant Pathology	TransferNet	The approach was designed to improve precision farming by providing apple producers with tech-driven diagnostic tools.	91.63%
Lin (2024)	Plant Village	ResNet-101	The Convolutional Block Attention Module uses ResNet-101 as the underlying network structure, improving the model’s accuracy and resilience.	96.69%
Banarase & Shirbahadurkar (2024)	Plant Village	MobileNet V2	The authors suggested adjusting the hyperparameters and training the MobileNetV2 model using various combinations of optimizers to attain the highest accuracy.	99.36%

## Discussion

Recently, many scientific studies (Kotwal, Kashyap & Shafi, 2024; Sharma, Tripathi & Mittal, 2023; Chilakalapudi & Jayachandran, 2024) have been proposed to classify and detect various plant leaf diseases. These studies have generally been carried out not for a single plant species but for multiple species. The valuable contribution of these studies to agriculture and producers is undeniable, but the production of customized datasets and models to obtain more successful models and results may make it possible for real applications to be accepted faster and by a wider target group. For this reason, apple, which has many different functions in the agricultural sector and is the most widely cultivated fruit, was chosen for this study.

Between 2016 and 2023, there is considerable growth in both conference and journal articles. Conference articles peaked at 35 in 2022, followed by journal articles at 28 in 2023. Over time, more conference articles were published than journal articles. This may indicate that in previous years, more practice-oriented aspects of the topic were discussed at conferences. The growth in the number of journal articles has accelerated, especially after 2021. This may indicate that academic studies are turning to journal publications for more in-depth analyses and long-term results. Data for 2024 are not yet complete, so the number of both conference (9) and journal (7) articles is lower than the previous year. However, we anticipate that by the end of the year this number may be higher than the previous year. The graph shows that an area such as apple leaf disease detection is gaining more and more attention and research in this field is increasing.

The datasets created by researchers with their individual efforts have the largest share with 35.56 percent. This shows that researchers tend to build custom datasets that allow them to address specific research questions that may not be covered by existing ones. In addition, creating custom data sets ensures that the data are up-to-date and directly aligned with the research objectives. Such a high proportion of self-created datasets reveals the need for originality and tailor-made data in this field. The Plant Village dataset was the second most widely used dataset. This indicates that this dataset is a widely known and trusted source. The Plant Pathology data set has a moderate usage rate. The Apple Research Institute dataset is the least used dataset, as it addresses a specific research area.

The datasets mentioned above are usually obtained from a single leaf or controlled conditions. Although the models built on these datasets show high-performance results, they cannot show the same success when used on the farm or under different conditions. For these reasons, datasets and models (Jiang et al., 2019; Singh et al., 2020) obtained under real conditions will be more useful to farmers in disease detection. Therefore, it is essential to increase similar studies and datasets. Collaboration between researchers and farmers is crucial to developing practical solutions that address real-world challenges. This also ensures that the technologies developed are both effective and readily adoptable by farmers, ultimately enhancing agricultural productivity and sustainability.

The framework that is selected to develop the models is equally important as the data set that is selected. When selecting a framework, it is crucial to take into account metrics such as community support, scalability, and simplicity of use. Performance and execution speed are essential factors, particularly for large-scale models. Keras and PyTorch are ideal for constructing categorization models because to their user-friendliness and compatibility with pretrained models. TensorFlow and PyTorch thrive because of their adaptability and endorsement of sophisticated object detection frameworks like as YOLO and Faster R-CNN. Matlab is an optimal selection for academic research and prototyping, particularly for individuals who want a GUI-driven interface. Caffe is extensively tuned for rapid performance and is appropriate for small-scale detection and classification jobs. PyTorch and TensorFlow are optimal selections for extensive apple leaf disease initiatives, particularly when integrating classification and detection inside a unified pipeline. Each platform possesses distinct advantages and applications, making it essential to select the one most appropriate for the intended usage.

**Figure 9:**
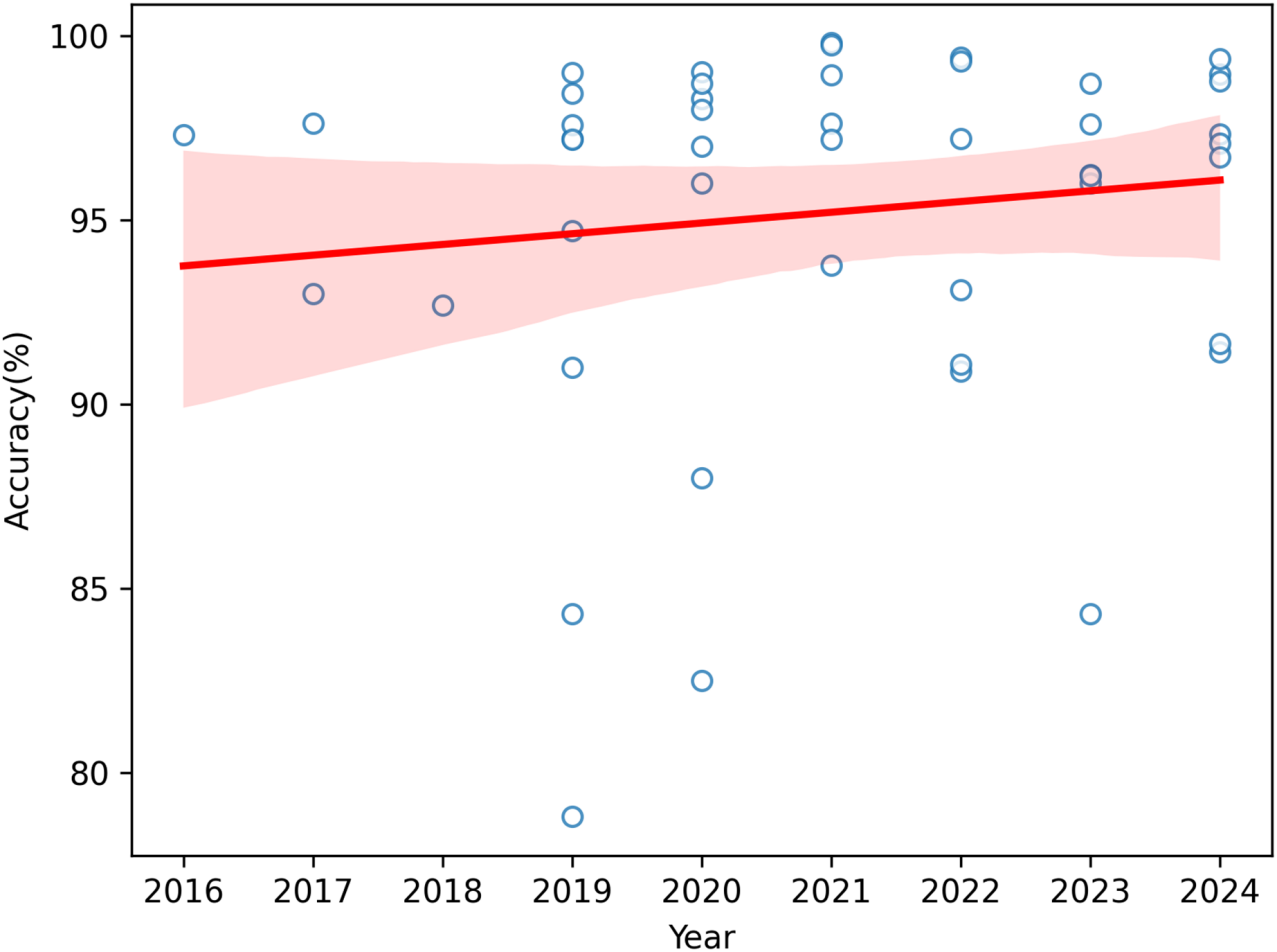
Distribution of DL frameworks for apple leaf disease detection and classification.

The accuracy values of the research that categorize and identify apple leaf diseases by year from 2016 to 2024 are displayed in Fig. 9. The regression line that predicts the association between accuracy and year is represented by the red line, while the model’s confidence interval is shown by the pink region. The graph shows that there is a general tendency toward greater accuracy. This pattern suggests that the models’ overall performance has become better over time.

## Conclusion

Artificial intelligence applications play a significant role in the classification and early detection of plant leaf diseases. This helps farmers and stakeholders improve overall yield production and reduce expenses for apple plants and other agricultural products. They can also provide accurate and timely plant disease predictions, enabling better resource management and avoiding potential economic losses. By comparing the performance of recent DL techniques to traditional and ML methods, this study shows that recent DL techniques are more effective in considering classification and detection. In addition, it presents details on 45 apple leaf disease detection studies, dataset sources, and contributions. It also recommends using the Apple dataset available from Plant Village rather than the self-datasets taken from unstable environments, resulting in a slower and less accurate disease identification process. The importance of creating datasets and models that reflect actual production conditions is again highlighted. To improve the real-world applicability of these models, researchers should focus on incorporating diverse environmental conditions into their datasets and collecting data from various geographical locations and seasons to ensure the models are robust under diverse circumstances. As such, this article provides a valuable basis for researchers to investigate and develop better disease classification and detection systems.

## Supplemental Information

10.7717/peerj-cs.2655/supp-1 Supplemental Information 1 PRISMA Checklist.

## Additional Information and Declarations

### Data Availability

The following information was supplied regarding data availability:

This is a literature review.
